# Sticky siRNAs targeting survivin and cyclin B1 exert an antitumoral effect on melanoma subcutaneous xenografts and lung metastases

**DOI:** 10.1186/1471-2407-13-338

**Published:** 2013-07-09

**Authors:** Valerie Kedinger, Aline Meulle, Omar Zounib, Marie-Elise Bonnet, Jean-Baptiste Gossart, Elodie Benoit, Melanie Messmer, Pattabhiraman Shankaranarayanan, Jean-Paul Behr, Patrick Erbacher, Anne-Laure Bolcato-Bellemin

**Affiliations:** 1 Polyplus-transfection SA, Bioparc, BP 90018, Boulevard Sébastien Brant, Illkirch, 67401, France; 2 Current address: IBMC, 15 rue René Descartes, Strasbourg, 67084, France; 3 IGBMC, 1 rue Laurent Fries, Illkirch, 67400, France; 4 Current address: Quintiles, rue Jean Dominique Cassini, BP 50137, Illkirch Cedex, 67404, France

**Keywords:** Sticky siRNA, Delivery, Survivin, Cyclin B1, Tumor inhibition, Melanoma

## Abstract

**Background:**

Melanoma represents one of the most aggressive and therapeutically challenging malignancies as it often gives rise to metastases and develops resistance to classical chemotherapeutic agents. Although diverse therapies have been generated, no major improvement of the patient prognosis has been noticed. One promising alternative to the conventional therapeutic approaches currently available is the inactivation of proteins essential for survival and/or progression of melanomas by means of RNA interference. Survivin and cyclin B1, both involved in cell survival and proliferation and frequently deregulated in human cancers, are good candidate target genes for siRNA mediated therapeutics.

**Methods:**

We used our newly developed sticky siRNA-based technology delivered with linear polyethyleneimine (PEI) to inhibit the expression of survivin and cyclin B1 both *in vitro* and *in vivo*, and addressed the effect of this inhibition on B16-F10 murine melanoma tumor development.

**Results:**

We confirm that survivin and cyclin B1 downregulation through a RNA interference mechanism induces a blockage of the cell cycle as well as impaired proliferation of B16-F10 cells *in vitro*. Most importantly, PEI-mediated systemic delivery of sticky siRNAs against survivin and cyclin B1 efficiently blocks growth of established subcutaneaous B16-F10 tumors as well as formation and dissemination of melanoma lung metastases. In addition, we highlight that inhibition of survivin expression increases the effect of doxorubicin on lung B16-F10 metastasis growth inhibition.

**Conclusion:**

PEI-mediated delivery of sticky siRNAs targeting genes involved in tumor progression such as survivin and cyclin B1, either alone or in combination with chemotherapeutic drugs, represents a promising strategy for melanoma treatment.

## Background

Melanoma is considered as one of the most aggressive human cancers. The majority of melanoma-associated deaths are caused by metastases, which can occur in a wider variety of areas than any other cancer [1]. Among target organs, the lung represents a common site of metastasis, mostly because of its anatomic structure and high vascularization. These characteristics make it a preferential pathway for metastatic seeding and a rich environment for neoplastic growth [2]. Another feature commonly attributed to melanoma is its chemo-resistance [3]. During melanoma progression, the breakdown of cell death control leading to resistance to chemotherapeutic drugs is achieved through the combined activation of anti-apoptotic factors, inactivation of pro-apoptotic effectors and reinforcement of survival signals. Targeting one or more of these different factors may be a key requisite to overcome drug resistance and thus improve clinical outcome of patients with melanoma.

Survivin, a member of the Inhibitor of Apoptosis Protein (IAP) family [4], has emerged few years ago as a promising therapeutic target in cancers because of its overexpression in a wide spectrum of tumors, including melanoma [5-7]. Additionally, survivin was identified as a marker of poor prognosis in melanoma [8]. In addition to its role in apoptosis inhibition, survivin also plays a critical role in the regulation of cell division by inducing exit from G1 checkpoint arrest and subsequent entry into S phase [9]. Finally, recent studies have involved survivin in cell motility, which may underlie a role for this protein in promoting melanoma metastasis [10-12]. Various approaches involving molecular inhibitors have been developed to inhibit its expression in tumor cells [6,13-17]. Another potential therapeutic target for cancer treatment is represented by cyclin B1, the regulatory subunit of cyclin-dependent kinase 1 (cdk1), which plays a pivotal role in the transition of the cell cycle from G2 phase to mitosis [18]. Altered expression of cyclin B1 has been reported in numerous cancers, where it could contribute to chromosomal instability [19-23]. Furthermore, several studies have demonstrated its clinical significance as a poor prognosis factor for several cancer types [24-27], including melanoma [28], and cyclin B1 overexpression is responsible for radiotherapy resistance in different tumors [29-31].

RNA interference represents a powerful approach for antitumor therapy by allowing *in vivo* silencing of essential genes for tumor progression and provides a promising alternative to traditional small molecule therapies. However, delivery of siRNAs still remains the most challenging step for the development of a siRNA-based therapy. The challenge includes efficient target gene silencing in the desired tissue while avoiding side effects such as immune response, toxicity and off-target silencing. In this context, the cationic linear polyethylenimine (PEI) is well known for its efficiency to transfect genes both *in vitro* and *in vivo* as it is involved in several clinical trials for the treatment of bladder cancer (http://clinicaltrials.gov/ct2/show/NCT00595088), pancreatic ductal adenocarcinoma (http://clinicaltrials.gov/ct2/show/NCT01274455) and multiple myeloma (http://clinicaltrials.gov/ct2/show/NCT01435720?term=senesco&rank=1). In this study, we investigated its ability to deliver functional anti-tumoral siRNA. To this end, we have developed sticky siRNAs (ssiRNAs) that mimic gene structure through reversible concatemerization brought by sticky 3’-complementary overhangs [32]. These modified siRNAs confer a higher stability to the complexes formed with linear PEI, thus increasing gene silencing efficiency both *in vitro* and *in vivo*, compared with standard siRNAs. We used this new technology to specifically target survivin and cyclin B1 in B16-F10 murine melanoma cells. Our results show that ssiRNAs are efficient to inhibit survivin and cyclin B1 expression *in vitro* and that a systemic treatment with ssiRNAs targeting these two genes is able to reduce both subcutaneous melanoma tumors and their lung metastases. Moreover, inhibition of survivin expression increased the effect of a doxorubicin treatment on melanoma lung metastasis. Altogether, our data are promising towards development of ssiRNAs against survivin and cyclin B1 as a new therapeutic strategy for melanoma treatment.

## Methods

### Cell line

Murine melanoma B16-F10 cell line was obtained from ATCC and cultured in Dulbecco’s modified Eagle’s medium (Eurobio, Courtaboeuf, France), supplemented with 10% fetal bovine serum (Hyclone, Logan, UT, USA), 2 mM Glutamine (Eurobio) and 200 U/ml penicillin / 200 μg/ml streptomycin (Eurobio).

### Sticky siRNAs

IEX-HPLC-purified nucleic acids were purchased from Eurogentec (Brussels, Belgium). Annealing was performed in annealing buffer (Eurogentec), final concentration 0.1 X for 2 min at 95°C followed by slow cooling. Sequences were as follow:

Cyclin B1 ssiRNA sense, 5’-GAGAUGUACCCUCCAGAAAdTdTdTdTdTdTdTdT-3’,

Cyclin B1 ssiRNA antisense, 5’-UUUCUGGAGGGUACAUCUCdAdAdAdAdAdAdAdA-3’,

Survivin ssiRNA sense, 5’-CCGUCAGUGAAUUCUUGAAdTdTdTdTdTdTdTdT-3’,

Survivin ssiRNA antisense, 5’-UUCAAGAAUUCACUGACGGdAdAdAdAdAdAdAdA-3’,

Negative control ssiRNA sense, 5’-AUGUCUACUGGCAGUCCUGdTdTdTdTdTdTdTdT-3’,

Negative control ssiRNA antisense, 5’-CAGGACUGCCAGUAGACAUdAdAdAdAdAdAdAdA-3’.

### In vitro and in vivo transfections

jetPEI® and *in vivo*-jetPEI® were from Polyplus-transfection (Illkirch, France). For transfection with jetPEI® reagent, complexes were prepared as follows: for a triplicate experiment, the required amount of ssiRNA and transfection reagent were each separately diluted in 150 μl of NaCl 150 mM. A volume of 2.4 μl (for 75 nM of ssiRNA, N/P=6) or 3.2 μl (for 50 nM of ssiRNA, N/P=8) of jetPEI® was used per μg of siRNA. N/P ratio is defined as the number of nitrogen residues of jetPEI® per nucleic acid phosphate. The transfection reagent solution was added to the ssiRNA solution and left for 30 min at room temperature. A volume of 100 μl of complexes was added to B16-F10 cells seeded in 24-well plates at 40,000 cells/well one day before, and placed in 0.5 ml of medium without serum just prior to complexes addition. After 4 h, the medium was replaced by 1 ml of complete medium containing 10% serum.

For *in vivo* delivery with *in vivo*-jetPEI®, complexes were prepared as follows: for 1 mouse, the required amount of nucleic acid and PEI were separately diluted in 100 μl of 5% glucose solution (final concentration). For injection of 0.6 mg/kg of ssiRNA, 0.16 μl of PEI were used per μg of ssiRNA (N/P=8). For the 1 mg/kg ssiRNA injected amount, 0.25 μl of PEI were used per μg of ssiRNA (N/P=12.5). The transfection reagent solution was added to the ssiRNA solution and left for at least 30 min at room temperature. At this stage complexes are stable for more than 4 h at room temperature.

### Animal experiments

All animal studies were conducted in accordance to the French Animal Care guidelines and the protocols were approved by the Direction des Services Vétérinaires. Five-weeks old NMRI Nude female mice were obtained from Elevage Janvier (Le Genset Saint Isle, France). For subcutaneous xenografts, B16-F10 cells (1 × 10^6^ cells in 100 μl of culture medium without serum) were injected subcutaneously on the right flank of animals. ssiRNA/PEI complexes were intravenously injected through the retro-orbital sinus within 2 s. Treatment with ssiRNA complexes started when tumors reached 50 mm^3^ and were performed every other day until sacrifice of the animals. Tumors were measured at each injection, and tumor volume was calculated as *v* = (π × L × l^2^)/6. For lung metastasis model, B16-F10 cells (1 × 10^6^ cells in 300 μl of culture medium without serum) were injected intravenously through the tail vein.

### Branched DNA assay

QuantiGene assay (Panomics, Santa Clara, CA, USA) was used to quantify the amount of mRNA in cells or lungs. Cells were lysed in 600 μl of 1 × lysis buffer and incubated for 30 min at 50°C. Lungs were lysed in 20 ml of tissue and cell lysis solution (Tebu, Le Perray-en-Yvelines, France), supplemented with 0.15 mg/ml of K Proteinase (Sigma-Aldrich, St Louis, MO, USA) and incubated three times 5 min at 60°C with 10 s vortexing. A volume of 1–60 μl of cell or lung lysate was used for branched DNA (bDNA) assay. Probe set were designed using QuantiGene ProbeDesigner software. Target gene expression was assayed according to manufacturer recommendations. Target expression level was normalized to corresponding GAPDH expression from the same cell lysate.

### Western blot analysis

Cells were lysed in 100 μl of RIPA buffer. Proteins were quantified with the BCA kit (Pierce, Brebieres, France). Fifty micrograms of total protein were subjected to electrophoresis on a 10 or 15% acrylamide/bisacrylamide gel and transferred to a poly (vinylidene fluoride) membrane (Millipore, Molsheim, France). A mouse anti-cyclin B1 monoclonal antibody (Cell Signaling Technologies, Danvers, MA, USA) at 1/1,000, a rabbit anti-survivin polyclonal antibody (Cell Signaling Technologies) at 1/1,000 and a mouse anti-GAPDH monoclonal antibody (Ambion, Austin, TX) at 1/10,000 were used. Anti-rabbit or anti-mouse secondary horseradish peroxidase-conjugated were purchased from Millipore and used at 1/10,000. Protein bands were visualized with enhanced chimioluminescence reagent (ECL, Amersham, GE Healthcare, Velizy-Villacoublay, France).

### Proliferation assay

Cell pellets were homogenized in 100 μl of 1:1 PBS/trypan blue (Eurobio) and live cells were counted using an automatic hematocyter (TC10, BioRad, Marnes-la-Coquette, France).

For nuclei morphology analysis, cells were fixed with ice-cold methanol for 10 min, rinsed with 1 × PBS and stained with DAPI (0.01 μg/μl) for 15 min. Cells were observed using a Nikon inverted microscope (Nikon Eclipse TE 2000-S, Amsterdam, Netherland).

### Cell cycle and apoptosis analysis

Cells were fixed in chilled 50% ethanol for 15 min at −20°C. Pellets were incubated in 1 × PBS with 0.1% Triton X-100 and 5% BSA for 10 min on ice, and for 30 min at 37°C in 1 × PBS containing 20 μg/ml of RNase A. Propidium iodide (100 μg/ml) was added for 10 min at room temperature. Cell pellet was resuspended in 1 × PBS, 5 mM EDTA to obtain a cell concentration lower than 5.10^5^ cells/ml. Cells were analyzed by FACS using a Guava apparatus from Millipore (Molsheim, France).

### 5’-RACE analysis

Total RNA was isolated using RNA NOW reagent (Biogentex Laboratories, Houston, TX, USA) following instructions of the manufacturer. One μg of RNA was ligated to GeneRacer ™ RNA Oligo (5’-CGA-CUG-GAG-CAC-GAG-GAC-ACU-GAC-AUG-GAC-UGA-AGG-AGU-AGA-AA-3’; Life Technologies, Saint Aubin, France). Two-hundred and fifty nanograms of Oligo were used respectively for cyclin B1 or survivin analysis. Ligated RNA was reverse transcribed using a gene-specific primer (Table 1). To detect the cleavage product, one or two rounds of consecutive PCR (for conditions see Additional file 1: Table S1) were performed using primers complementary to the RNA Oligo and to cyclin B1 or survivin gene sequence (Table 2).

**Table 1:** Primers used for Reverse transcription

**Name**	**Sequence**
Cyclin B1 Rev (CCNBM1548R)	5′-TTC-GAC-AAC-TTC-CGT-TAG-CC-3′
Survivin Rev (810R)	5′-AGC-TCT-GGA-CTC-TGG-CCA-CCC-3′

**Table 2:** Primers used for PCR

**Name**	**Sequence**
Cyclin B1 Rev (CCNBM975R)	5′-AGG-GCG-ACC-CAG-GCT-GAA-GT-3′
Survivin Rev (810R)	5′-AGC-TCT-GGA-CTC-TGG-CCA-CCC-3′
Survivin RevN (743R)	5′-GCC-ACC-TCC-CTG-TGG-ACT-CA-3′

### Histology

After mice sacrifice, lungs were perfused with 4% paraformaldehyde, incubated for 16 h in 4% paraformaldehyde and processed for paraffin embedding. Paraffin sections (7 μm) were generated and Hematoxylin/Eosin (H&E) stained.

### Statistical analysis

All statistical analyses were performed using GraphPad prism software package (version 5). P values were calculated according to Mann-Withney test and were considered significant when lower than 0.05.

## Results and discussion

### in vitro ssiRNAs gene silencing efficiency in B16-F10 murine melanoma cells

**Figure 1:**
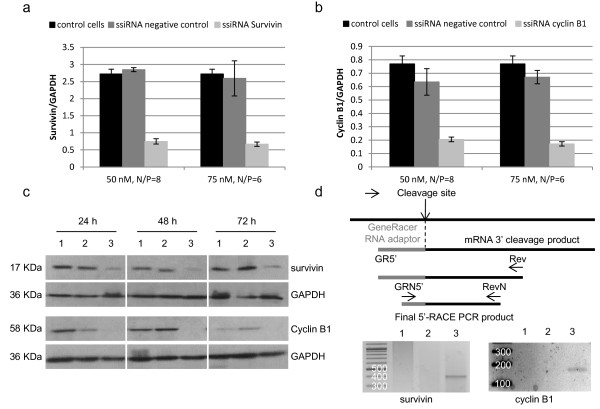
**Inhibition of survivin and cyclin B1 expression in B16-F10 cells. (a, b)** mRNA expression level of cyclin B1 and survivin analyzed by branched DNA 16 h after transfection with ssiRNAs and PEI. Cyclin B1 and survivin levels are expressed relative to GAPDH. **(c)** Protein level of cyclin B1 and survivin analyzed by Western blot 24, 48 and 72 h after transfection with a negative control ssiRNA (2) or ssiRNAs against cyclin B1 or survivin (3) (50 nM, N/P=8). Non transfected cells (1) were also loaded. GAPDH was used as a loading control. **(d)***In vitro* 5’-RACE analysis of RNA extracted from B16-F10 cells either untransfected (1) or 16 h after transfection with negative control (2), cyclin B1 or survivin (3) ssiRNAs and PEI (50 nM, N/P 8). Top part, schematic representation of the 5’-RACE procedure.

We used previously validated survivin and cyclin B1 siRNA sequences [33,34], designed sticky siRNAs with these sequences by adding 3’ complementary overhangs and first tested their silencing efficiency both at the mRNA and protein levels in B16-F10 cells. A sequence presenting no homology with mRNA databases was used as a negative control. Transfection of B16-F10 cells with jetPEI® and different concentrations of specific ssiRNAs ranging from 25 to 100 nM was performed and all led to a significant reduction of cyclin B1 and survivin mRNA levels. The level of inhibition was optimal at 50 nM, N/P of 8 and 75 nM, N/P of 6 (Figure 1a and b). Indeed, both concentrations induced a 70 to 80% inhibition of the two target mRNA expression compared to control cells. This downregulation of mRNA expression induced a diminution of the corresponding protein level for at least 48 h for both target proteins, as analyzed by Western blot (Figure 1c).

In order to confirm that the gene silencing observed after transfection occurred through a RNAi mechanism, we then performed rapid amplification of cDNA ends (5’-RACE) on RNAs extracted from B16-F10 cells transfected with cyclin B1, survivin or negative control ssiRNA. A band corresponding to the predicted cleavage product (158 bp for cyclin B1 and 330 bp for survivin) was specifically detected from RNAs extracted of both cyclin B1 and survivin ssiRNA transfected cells (Figure 1d). Sequencing analysis of the PCR products confirmed their specificity (data not shown).

**Figure 2:**
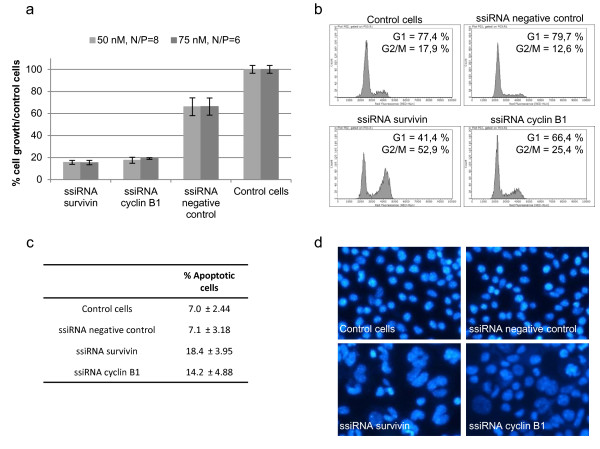
**Effect of survivin and cyclin B1 inhibition on B16-F10 cell cycle progression*. *(a)** Proliferation assay 48 h after transfection of ssiRNAs. The proliferation rate is represented as a percentage of cell growth relative to control untransfected cells. **(b)** Flow cytometer analysis of the cell cycle distribution of untransfected B16-F10 cells or 48 h after transfection with negative control, survivin or cyclin B1 ssiRNAs with PEI (50 nM, N/P=8). **(c)** Table presenting the percentage of cells in apoptosis (subG1 population) of 3 independent experiments determined after FACS analysis in the presence of propidium iodide 48 h after transfection with negative control and specific ssiRNAs with PEI (50 nM, N/P=8). **(d)** DAPI staining, 48 h after transfection with negative control, cyclin B1 or survivin ssiRNAs with PEI (50 nM, N/P=8) showing the presence of mega-nuclei and chromosomal aberrations in cells transfected with either cyclin B1 or survivin ssiRNAs.

The inhibition of cyclin B1 and survivin expression observed after ssiRNA transfections was accompanied by a specific cellular effect, as cells presented a growth inhibition of more than 80% compared to control cells 48 h post-transfection (Figure 2a). The proliferation rate of cells transfected with the negative control ssiRNA was also somewhat lowered compared to control cells. This diminution can be attributed to the transfection itself, which causes a transient blockage of the cell cycle progression. To further characterize the growth inhibition induced by the down-regulation of both cyclin B1 and survivin, we analyzed by flow cytometry B16-F10 DNA content 48 h after transfection of ssiRNAs (Figure 2b). Whereas only 17.9 and 12.6% of nontransfected or negative control ssiRNA-transfected cells were located in G2/M phase, respectively, more than 50% of cells transfected with survivin ssiRNA were arrested in G2/M phase. Comparatively, the effect was milder after cyclin B1 inhibition, yet a significant blockage of the cells in G2/M phase was still present (Figure 2b). Moreover, percentage of apoptotic cells was also increased following downregulation of survivin and cyclin B1 expression compared to controls (Figure 2c). Finally, DAPI staining of cells in which cyclin B1 or survivin expression was inhibited showed a high proportion of cells presenting mega-nuclei (more than 50% of the cells), a characteristic of cell cycle arrest (Figure 2d).

Altogether, these results validate the ssiRNA approach to silence cyclin B1 and survivin through a mechanism of RNA interference *in vitro* in B16-F10 murine melanoma cells.

### Systemic treatment with ssiRNAs inhibits growth of established subcutaneous melanoma xenografts

**Figure 3:**
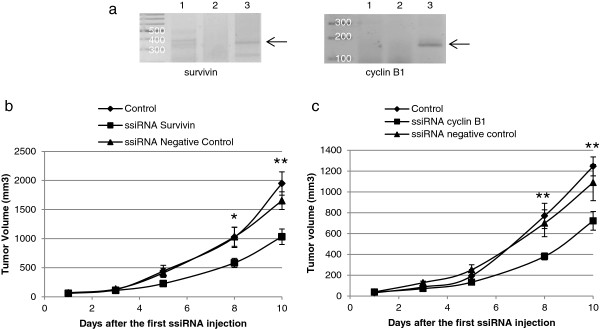
**B16-F10 tumor xenograft growth inhibition upon systemic treatment of mice with ssiRNAs and PEI. (a)***In vivo* 5’-RACE analysis of RNA extracted from subcutaneous tumors 16 h after intra-tumoral injection of 0.6 mg/kg of negative control (2) and survivin or cyclin B1 (3) ssiRNA and PEI (N/P=8). As a control, 5’-RACE was also performed on RNA extracted from a tumor injected with glucose only (1). **(b, c)** Mice bearing tumor xenografts were treated intravenously every other day with glucose (diamond), negative control ssiRNA (triangle) and survivin **(b)** or cyclin B1 **(c)** ssiRNA (square) at 1 mg/kg, N/P=12.5. Tumor growth was monitored after each ssiRNA injection and represented as a mean tumor volume ± SEM. n = 10 animals per group. P values were calculated between control (glucose treated) group and specific ssiRNA groups; * *p*<0.05; **p<0.01.

B16-F10 cells have the ability to form tumors when injected subcutaneously in nude mice [35]. We took advantage of this model mimicking a primary melanoma tumor to test the potency and specificity of PEI-mediated delivery of ssiRNAs *in vivo*. First, in order to confirm a RNA interference mechanism of ssiRNAs *in vivo*, we performed a 5’-RACE analysis after intra-tumoral injection of ssiRNA (0.6 mg/kg, N/P=8) complexed with *in vivo*-jetPEI®. As shown in Figure 3a, we detected a band specific for the cleavage product after injection of either cyclin B1 or survivin ssiRNAs in B16-F10 xenografts that could not be detected in glucose or negative control ssiRNA injected tumors.

We then assessed the effect of a systemic treatment of ssiRNAs delivered with *in vivo*-jetPEI® on the growth of B16-F10 xenografts. Intravenous delivery of ssiRNAs at 1 mg/kg (N/P = 12.5) was performed every other day. The mean tumor size monitored after each injection is represented in Figure 3b for each group. We started to observe a reduction of tumor growth after the third injection of ssiRNA against survivin (i.e., 5 days after tumor cells injection), which persisted until the end of the experiment and reached a significant inhibition of up to 50% compared to control mice (Figure 3b). The effect of cyclin B1 ssiRNA was less pronounced, yet still significant as it leads to 44% inhibition of tumor growth compared to controls (Figure 3c). The difference observed between the two target genes could be explained by the multifunctionality of survivin compared to cyclin B1, as the first protein is implicated in apoptosis, proliferation and motility [36], whereas the second one only plays a critical role in the control of the cell cycle progression and apoptosis [18]. The mice treated with negative control ssiRNA presented a minor, statistically nonsignificant, reduction of tumor size. The difference between the control and negative control ssiRNA groups could reflect the nonspecific effect of transfection on the cell cycle previously observed *in vitro* (see above).

### Melanoma lung metastases are reduced following systemic treatment with ssiRNAs

**Figure 4:**
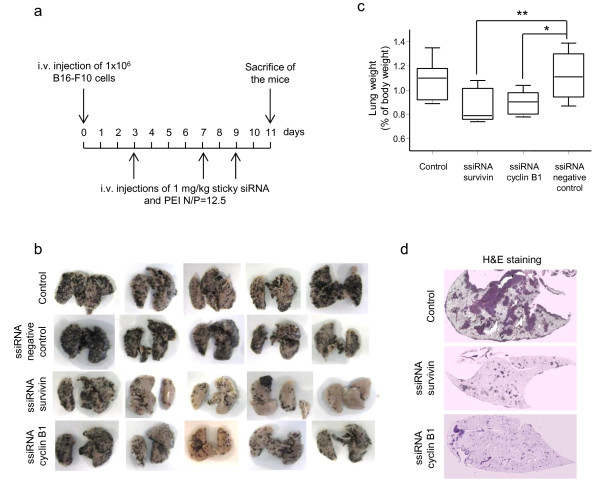
**Anti-metastatic therapeutic effect of a systemic treatment with ssiRNAs and PEI.** Nude mice were i.v. injected with B16-F10 cells, randomized and treated with glucose, negative control, survivin or cyclin B1 ssiRNAs and PEI (1 mg/kg, N/P=12.5). **(a)** Schematic representation of the treatment procedure. **(b)** Representative pictures of lungs excised from mice of the different treated groups. **(c)** Overall mean lung weight relative to the body weight of the mice. n = 8 animals per group. P values were calculated; * *p*<0.05; **p<0.01. **(d)** Representative images of Hematoxylin-Eosin stained lung sections after treatment with glucose, survivin or cyclin B1 ssiRNAs and PEI.

Metastases, which represent the leading cause of mortality for patients with melanoma, are the most challenging cells to target. PEI was previously shown to preferentially deliver active nucleic acids, plasmids and siRNAs, to the lung after intravenous injection [37-40]. We therefore wondered whether systemic treatment of antitumoral ssiRNAs delivered with PEI could reduce the formation of melanoma lung metastases. When injected into nude mice tail vein, B16-F10 cells rapidly give rise to lung metastases. The advantage of this model is that it circumvents the initial step of tumor cell migration and allows us to evaluate the effect of our treatment on the implantation of metastatic cells in the host tissue and their subsequent proliferation. Treatment with ssiRNAs was performed as described in Figure 4a. At the end of the experiment, lungs were excised and observed for metastatic nodules. Treatment with cyclin B1 and survivin specific ssiRNAs led to an important reduction of the number of metastases (black nodules) compared to controls as illustrated by representative pictures of lungs (Figure 4b). The lung weight was also used as an indicator of metastasis progression. We determined the mean percentage of lung over body weight in each group and observed a significant decrease in groups treated with survivin and cyclin B1 ssiRNAs compared to control groups (Figure 4c). Histological analysis of lung sections was performed which further confirmed the drastic diminution of both nodule size and number in both survivin and cyclin B1 ssiRNA treated lungs (Figure 4d).

**Figure 5:**
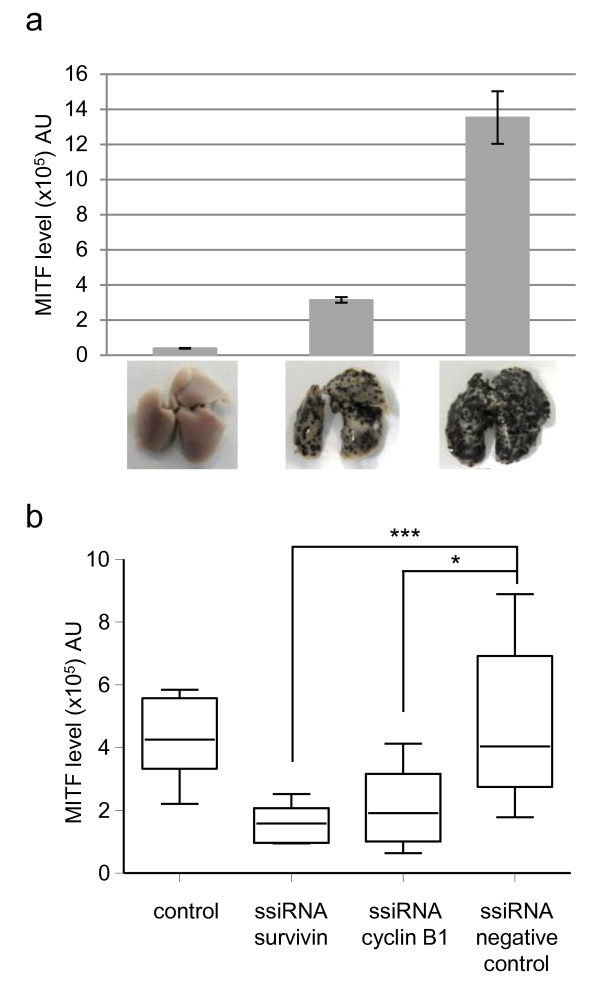
**Quantification of the B16-F10 lung metastases using dosage of MITF melanocyte specific transcription factor expression*. *(a)** Branched DNA analysis of RNA extracted from wild-type or B16-F10 tumor-bearing lungs for determination of MITF transcription factor mRNA expression level. **(b)** MITF RNA level was analyzed by branched DNA in B16-F10 tumor-bearing lungs untreated (control) or after a systemic treatment with survivin and cyclin B1 or negative control ssiRNA delivered with *in vivo*-JetPEI® (1 mg/kg, N/P=12.5). n = 6 to 9 animals per group. P values were calculated; * *p*<0.05; ***p<0.001.

Even if representative of the metastatic grade, lung weight evaluation is not the best quantitative method, and probably leads to an underestimation of the effect produced by the ssiRNAs injection. Indeed, with this method only 20 and 25% reduction of lung tumor weight were observed following cyclin B1 and survivin ssiRNA treatment, respectively. We thus developed a quantitative assay based on MITF dosage. Indeed, as MITF is a melanocyte specific transcription factor [41], it allows quantification of the B16-F10 nodules present in the lungs. When MITF expression is detected in lungs, its level should be directly proportional to the number of B16-F10 tumor cells. To verify this hypothesis, we analyzed MITF mRNA level in lungs containing increasing number of B16-F10 tumors (Figure 5a). As expected, no MITF mRNA was observed in lungs without B16-F10 tumors. In lungs presenting an intermediate number of B16-F10 tumors, an intermediate level of MITF was observed, whereas in lungs with a high level of tumor cells, a high level of MITF was observed (Figure 5a). In addition, we validated the linearity of our MITF assay (see Additional file 2: Figure S1).

We then looked at the MITF mRNA level in lungs of ssiRNA-treated mice, and observed a significant diminution after either survivin or cyclin B1 ssiRNA systemic treatment compared to the level of MITF present in lungs of glucose or negative control ssiRNA treated mice (65 and 56% inhibition compared to negative control ssiRNA, respectively, Figure 5b). These results are in better agreement with our macroscopic and microscopic observations, and confirm the anti-metastatic effect of a systemic treatment of mice with ssiRNAs targeting genes implicated in the cell cycle regulation.

### Alternated treatment with doxorubicin and survivin ssiRNA induces an additive diminution of melanoma lung metastases

**Figure 6:**
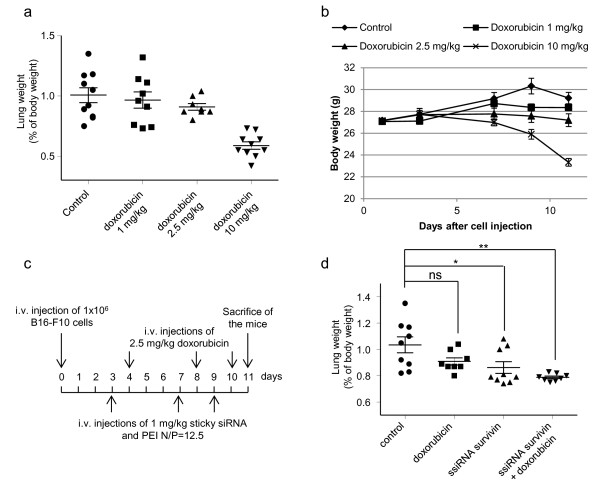
**Additive effect of survivin ssiRNA and doxorubicin on the treatment of lung metastases. (a)** Dose dependent effect of doxorubicin (1, 2.5 or 10 mg/kg) on lung metastasis growth assessed by the overall mean lung weight relative to the body weight of the mice. n = 9 animals per group. **(b)** Body weight measurement from the beginning to the end of the treatment was evaluated to determine the toxicity of doxorubicin treatments. **(c)** Schematic representation of the treatment procedure with doxorubicin alone, survivin ssiRNA alone, or both. **(d)** Overall mean lung weight relative to the body weight of the mice after an alternated treatment of doxorubicin and survivin ssiRNA with PEI, compared to treatment with doxorubicin or survivin ssiRNA alone. n = 9 animals per group. P values were calculated; * *p*<0.05; **p<0.01.

The poor prognosis attributed to melanomas largely results from resistance to conventional chemotherapy [3,42]. Doxorubicin, a topoisomerase II inhibitor, has been used for years to treat diverse types of cancers, and it is one of the most effective anticancer drugs currently known. However, its clinical use is limited by dose-dependent toxicity, low specificity against cancer cells and emergence of resistance [43]. Indeed, melanoma tumors are known to be partially refractory to doxorubicin [3], and high doses with subsequent toxic effects are necessary to induce tumor regression. Since survivin was shown to be a key factor in chemo-resistance, we hypothesized that a treatment with survivin ssiRNA could enhance the effect of doxorubicin on the inhibition of melanoma lung metastasis growth. In order to answer this question, we first established the optimal dose of doxorubicin, which would induce tumor growth inhibition with the least toxicity. To this end, three doxorubicin treatments (at day 4, 8 and 10) at three different doses (1, 2.5 and 10 mg/kg) were performed following B16-F10 tumor cells injection. Tumor growth inhibition was evaluated by lung weight and toxicity was determined by body weight loss during the course of the experiment. The treatment with doxorubicin at 1 mg/kg was not toxic at all but had no effect on tumor growth either. On the other end, the 10 mg/kg dose was very efficient on lung tumors but induced a drastic body weight loss, almost 15% at the end of the experiment, accompanied by a general posture of the mice which was characteristic of a high toxicity (Figure 6a and b). We thus retained the intermediate dose of 2.5 mg/kg which had only a mild effect on tumor growth inhibition but also shows minimal toxicity for the animals. We then evaluated the effect of an alternating treatment of doxorubicin and survivin ssiRNA compared to a treatment with either doxorubicin or survivin ssiRNA alone. The treatment with doxorubicin and ssiRNA was performed every alternate day as illustrated on Figure 6c. Doxorubicin (2.5 mg/kg) and survivin ssiRNA (1 mg/kg) had an additive effect on lung metastasis growth inhibition (Figure 6d). Whereas doxorubicin alone at 2.5 mg/kg was poorly efficient, its combination with survivin ssiRNA induced a significant metastasis inhibition as determined by a lung weight decrease compared to controls, and this without producing any toxicity. A strong diminution of the number and size of tumor nodules was observed visually (data not shown). Moreover, dosage of MITF mRNA level showed a 87% inhibition of tumor growth (MITF level, 0.6 × 10^5^ +/− 0.06 × 10^5^) following the alternating treatment of doxorubicin and survivin ssiRNA compared to controls (MITF level, 4.3 × 10^5^ +/− 0.6 × 10^5^) which was significantly higher than the survivin ssiRNA conditions without doxorubicin (54% inhibition compared to control, MITF level, 1.9 × 10^5^ +/− 0.4 × 10^5^).

These results are very encouraging for the development of survivin ssiRNA as a new strategy to circumvent the inherent chemo-resistance of melanomas or to enhance the chemosensitivity of melanomas. This combination therapy using ssiRNAs and chemotherapy offers a novel strategy for cancer treatment and is confirmed in other cancers [44,45].

## Conclusion

RNA interference is evolving as a promising strategy for cancer treatment. However, delivery of siRNAs *in vivo* still remains the major issue for the development of a successful siRNA-based therapy. Our present study highlights an emerging RNAi technology, based on the use of sticky siRNAs delivered with PEI. These modified siRNAs, by mimicking gene structure, enhance siRNA delivery into cells and consequently lead to a better inhibition of genes involved in malignancies. In order to validate this new technology, we chose to design sticky siRNAs against two well-known players of the tumor progression process, survivin and cyclin B1 [19,46,47]. The results presented in this work demonstrate that PEI-mediated systemic delivery of sticky siRNAs against survivin and cyclin B1 lead to an efficient inhibition of tumor growth. Moreover, we showed in a previous study that no major inflammation is induced by linear PEI-mediated nucleic acid delivery *in vivo*, as neither pro-inflammatory cytokines nor hepatic enzymes were produced [48]. Altogether, these data are very encouraging for the clinical development of such therapies which could represent a promising approach for melanoma treatment.

## Abbreviations

ssiRNA: Sticky siRNA; PEI: Polyethyleneimine; N/P: PEI amine over nucleic acid phosphate charge ratio.

## Competing interests

All the authors except Pattabhiraman Shankaranarayanan are employees of the Polyplus-transfection company, they declare no competing interests.

## Authors’ contributions

VK carried out *in vivo* and *in vitro* experiments, participated in the design of the study and drafted the manuscript, AM carried out *in vivo* experiments and participated in the design of the study, OZ carried out *in vivo* and *in vitro* experiments, MEB carried out *in vivo* and *in vitro* experiments, JBG carried out *in vivo* and *in vitro* experiments, EB carried out *in vivo* experiments, MM carried out *in vitro* experiments, PS gave some material and critically revised the manuscript, JPB participated in the design of the study and critically revised the manuscript, PE conceived the study, coordinated and helped to draft the manuscript, ALBB conceived the study, participated in its design and coordinated and helped to draft the manuscript. All authors read and approved the final manuscript.

## Pre-publication history

The pre-publication history for this paper can be accessed here:

http://www.biomedcentral.com/1471-2407/13/338/prepub

## Supplementary Material

Additional file 1: Table S1 PCR conditions for 5’ RACE analysis. Click here for file

Additional file 2: Figure S1 Linearity of MITF assay. Branched DNA analysis of RNA extracted from wild-type or B16-F10 tumor-bearing lungs to determine MITF mRNA expression level. Different volumes of lung extract (0.1; 0.5; 1 and 5 μl) were analyzed, showing a good linearity of the assay. Click here for file
