# Supplementary material for: Sticky siRNAs targeting survivin and cyclin B1 exert an antitumoral effect on melanoma subcutaneous xenografts and lung metastases
Source: BMC Cancer. 2013 Jul 9;13:338. doi: 10.1186/1471-2407-13-338 (PMC3711931; doi:10.1186/1471-2407-13-338)
Supplement: Additional file 1: Table S1 — PCR conditions for 5’ RACE analysis. [file 1471-2407-13-338-S1.ppt]

## Slide 1
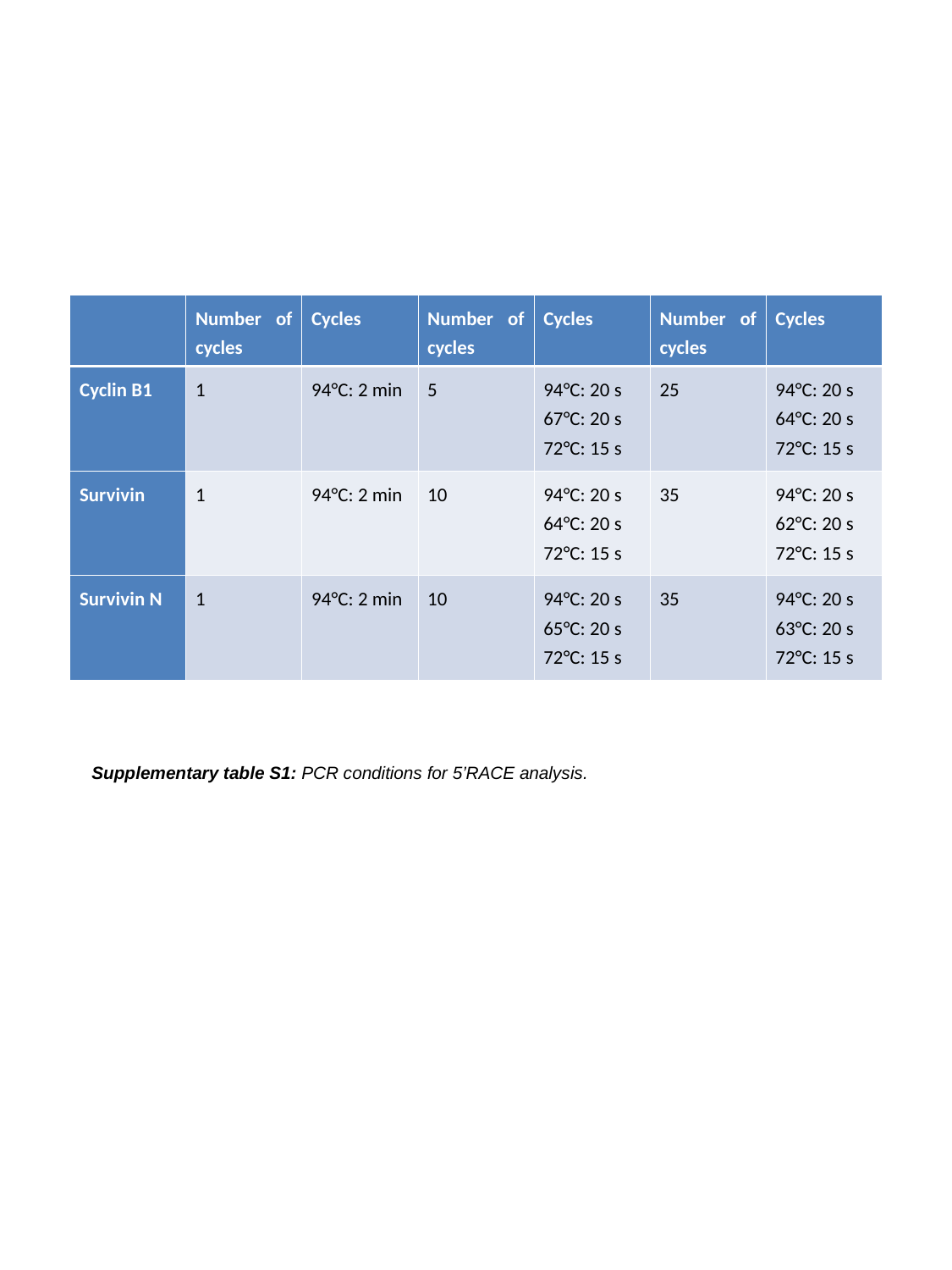

| | Number of cycles | Cycles | Number of cycles | Cycles | Number of cycles | Cycles |
| --- | --- | --- | --- | --- | --- | --- |
| Cyclin B1 | 1 | 94°C: 2 min | 5 | 94°C: 20 s 67°C: 20 s 72°C: 15 s | 25 | 94°C: 20 s 64°C: 20 s 72°C: 15 s |
| Survivin | 1 | 94°C: 2 min | 10 | 94°C: 20 s 64°C: 20 s 72°C: 15 s | 35 | 94°C: 20 s 62°C: 20 s 72°C: 15 s |
| Survivin N | 1 | 94°C: 2 min | 10 | 94°C: 20 s 65°C: 20 s 72°C: 15 s | 35 | 94°C: 20 s 63°C: 20 s 72°C: 15 s |
Supplementary table S1: PCR conditions for 5’RACE analysis.
