# Supplementary material for: Sticky siRNAs targeting survivin and cyclin B1 exert an antitumoral effect on melanoma subcutaneous xenografts and lung metastases
Source: BMC Cancer. 2013 Jul 9;13:338. doi: 10.1186/1471-2407-13-338 (PMC3711931; doi:10.1186/1471-2407-13-338)
Supplement: Additional file 2: Figure S1 — Linearity of MITF assay. Branched DNA analysis of RNA extracted from wild-type or B16-F10 tumor-bearing lungs to determine MITF mRNA expression level. Different volumes of lung extract (0.1; 0.5; 1 and 5 μl) were analyzed, showing a good linearity of the assay. [file 1471-2407-13-338-S2.ppt]

## Slide 1
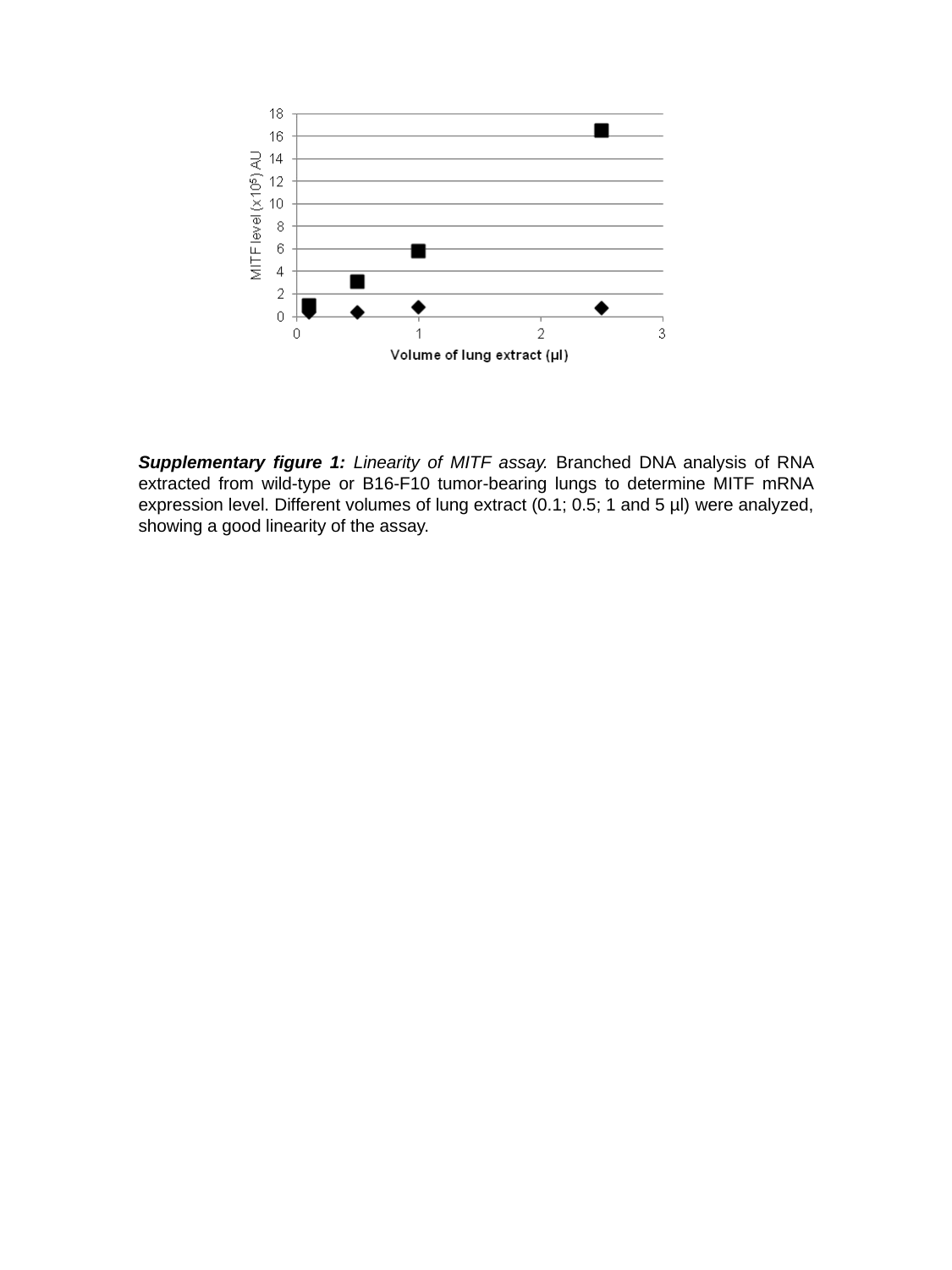

Supplementary figure 1: Linearity of MITF assay. Branched DNA analysis of RNA extracted from wild-type or B16-F10 tumor-bearing lungs to determine MITF mRNA expression level. Different volumes of lung extract (0.1; 0.5; 1 and 5 µl) were analyzed, showing a good linearity of the assay.
